# Editorial: Challenges in the contemporary assessment of coronary physiology

**DOI:** 10.3389/fcvm.2023.1305913

**Published:** 2023-10-12

**Authors:** Srdjan Aleksandric, Milorad Tesic, Dejan Orlic

**Affiliations:** ^1^ Cardiology Clinic, University Clinical Center of Serbia, Belgrade, Serbia; ^2^ Faculty of Medicine, University of Belgrade, Belgrade, Serbia

**Keywords:** fractional flow reserve, instantaneous wave-free ratio, pullback pressure gradient, machine learning, optical coherence tomography, transthoracic Doppler echocardiography, diastolic deceleration time, index of microvascular resistance

**Editorial on the Research Topic** Challenges in the contemporary assessment of coronary physiology

In the recently published Research Topic “Challenges in the Contemporary Assessment of Coronary Physiology” of Frontiers in Cardiovascular Medicine Journal, two review articles and three original studies were focused on the novel invasive and non-invasive image-based modalities for the assessment of coronary physiology. The physiological assessment of coronary artery disease (CAD) has become an integral part of the decision-making process for myocardial revascularization. There are currently more than 10 modalities available for the functional assessment of coronary stenosis severity, although these physiological tools, whether intracoronary or image-based, are still not widely adopted (1). In this Research Topic, Dobric at al. have presented current status and future perspectives of commercially available image-based fractional flow reserve (FFR) which is derived from invasive coronary angiography (CA), such as: (1) quantitative flow ratio (QFR; Medis Medical Imaging System, Leiden, the Netherlands and Pulse Medical Imaging Technology, Shanghai, China); (2) vessel FFR (vFFR; Pie Medical Imaging, Maastricht, the Netherlands); (3) FFRangio (CathWorks, Kefar Sava, Israel); (4) computational pressure-flow dynamics derived FFR (caFFR; FlashPressure, Rainmed Ltd, Suzhou, China); and (5) AccuFFRAngio (ArteryFlow Technology, Hangzhou, China). These angiography-derived indices could provide an FFR estimation using 3D reconstruction of the interrogated coronary arteries derived from at least two separated angiographic projections taken during invasive CA, without the use of drugs that induce hyperemia, a significantly prolongation of procedural time, patient discomfort, additional costs and the possibility of damaging the coronary artery with the wire (1). All of these technologies showed excellent diagnostic accuracy for detecting the invasive FFR ischemic values ≤0.80 with areas under the curves (AUCs) between 93% and 98%, and with a low incidence of non-analyzable cases (0.9%–10%) (1–11). These findings implicate that angiography-derived physiological indices without hyperemia could provide fast, simple and accurate identification of coronary lesions associated with inducible ischemia. However, only QFR was prospectively validated in a large randomised controlled trial (FAVOR III China trial) which compare the clinical outcomes of myocardial revascularization guided by QFR and revascularization guided by standard visual angiographic assessment (12). This trial showed that myocardial revascularization guided by QFR resulted in a 35% risk reduction of major adverse cardiac events (MACE) during one-year follow-up compared with standard visual angiography guidance (HR: 0.65; 95% CI: 0.51–0.83; *p*=0.0004), which was mainly driven by a lower rates of myocardial infarction (HR; 0.59; 95%CI: 0.44–0.81; *p*=0.0008) and ischaemia-driven revascularisation (HR: 0.64; 95% CI: 0.43–0.96; *p*=0.031), whereas mortality was similar between groups (HR: 1.44; 95% CI: 0.62–3.37; *p*=0.40). Moreover, QFR-guided revascularization led to fewer stents being used and less contrast and radiation exposure for patients (12). The ongoing trial (FAVOR III Europe Japan Study; NCT03729739) is currently investigating whether QFR-guided PCI is non-inferior to a standard invasive FFR-guided PCI in patients with stable angina and intermediate coronary stenosis regarding clinical outcomes at 1-year follow-up after the index procedure.

Furthermore, it is essential to mention the FFR derived from coronary computed tomography angiography (FFRct) which was developed using 3-dimensional reconstruction of the coronary arteries and computational fluid dynamics (13). The CT-derived FFR was also found to have a high diagnostic performance with AUCs between 90% and 93% for the detection and exclusion of ischemia-induced coronary lesions (14–16). However, both angiography-derived and CT-derived FFRs are questionable in patients with history of myocardial infarction, heart failure, aortic stenosis, the presence of a chronic total occlusion and other lesion subsets, such as left main, bifurcation and/or ostial lesions (1). Additionally, none of both angiography-derived and CT-derived FFRs have been validated against noninvasive functional tests (1). Further limitations of FFRct is the presence of motion artifacts and suboptimal imaging quality due to irregular heart rate and/or significant obesity (1).

In the other study, Cha et al. investigated for the first time the diagnostic accuracy of machine learning—fractional flow reserve (ML-FFR) based on optical coherence tomography (OCT) with invasive FFR in 356 coronary arteries from 130 patients with angiographically intermediate coronary lesions (40%−70% diameter stenosis). They devoleped an OCT-based ML algorithm using 7 major features with the best performance for the prediction of FFR ≤0.80: vessel type feature and 6 additional features from OCT image analysis [percent area stenosis, minimal lumen area (LA), lesion length, proximal LA, distal LA, and plaque area]. The key findings of the study were: (1) the OCT-based ML algorithm had a good diagnostic accuracy for predicting the invasive FFR ischemic values ≤0.80 (AUC 95%) in testing group (356 coronary lesions), with a sensitivity, specificity, and accuracy of 98%, 61% and 92%, respectively; (2) this ML algorithm had a lower diagnostic accuracy for predicting the invasive FFR ≤0.80 (AUC 91%) in the external validation group (101 coronary lesions) compared with the testing group, with a sensitivity, specificity and accuracy of 90%, 71% and 83%, respectively; and (3) the OCT-based ML algorithm could provide an FFR estimation within several minutes, which is a significant improvement over Computational Flow Dynamics (CFD) and Navier-Stokes equations, which are time-consuming and require extensive computation power, making it difficult to use such a system online during the procedure. In the recently published study by Huang et al. it has been found that OCT-based FFR named Optical Flow Ratio (OFR; OctPlus software v1.0, Pulse Medical Imaging Technology, Shanghai, China), was even superior to QFR in determining the functional significance of coronary lesion severity defined as invasive FFR ≤0.80, regardless of the presence of previous myocardial infarction and/or percutaneous coronary intervention (PCI) (17). The OFR had an excellent diagnostic accuracy for predicting the invasive FFR ischemic values ≤0.80 (AUC 97%), with a sensitivity, specificity and accuracy of 86%, 95%, and 92%, respectively; whereas diagnostic accuracy of QFR was lower (AUC 92%), with a sensitivity, specificity and accuracy of 88%, 87%, and 87%, respectively. Several advantages contribute to OCT-based FFR or OFR superiority over other imaging modalities (angiography, CT, intravascular ultrasound) in identifying functionally significant stenosis: (1) OCT provides high-resolution images with unprecedented spatial resolution allowing high-definition visualization of intraluminal and endothelial structures with more accurate lumen geometry than angiographic images; (2) OCT has low intra- and inter-observer variability, and therefore, is able to provide data without bias; (3) OFR is not dependent on angiographic projections; and (4) OFR can overcome inherent limitations of angiography and CT-based FFR, such as vessel foreshortening and overlap (17). Clinical trials comparing the impact of OCT-guided PCI with OFR vs. FFR-guided PCI on short- and long-term clinical outcomes would be clinically beneficial in the future.

In the comprehensive review article, Ilic et al. discussed in detail about current problems in the functional assessment of individual stenosis severity in serial coronary lesions. Prior studies have demonstrated that individual coronary stenosis may be underestimated in the presence of tandem or serial lesions during hyperemic physiological assessments (18). The main reason for this is that the hyperemic coronary blood flow (CBF) across one stenosis is affected by a second or serial stenosis, resulting in hemodynamic interdependence or “cross-talk” phenomenon between coronary stenoses, such that a proximal stenosis affects the FFR of a distal stenosis, and vice versa (19). Theoretically, it was assumed that nonhyperemic physiological index—instantaneous wave-free ratio (iFR) is immune to hemodynamic interdependence in serial stenoses under resting conditions, because coronary autoregulation keeps resting CBF stable as long as the lesion severity is mild to intermediate, preventing hemodynamic interaction between serial stenoses. Conversely, recently published studies have demonstrated that iFR is also affected by hemodynamic interdependence in the resting state and that individual stenosis severity is underestimated when serial disease is present (18–20). This “cross-talk” phenomenon is particularly pronounced in more severe stenoses. Yet, it remains unclear whether resting or hyperemic pullback-guided PCI in the presence of serial stenoses is superior to the other regarding short- and long-term clinical outcomes.

To better characterize patterns of CAD in one artery, Collet et al. proposed a new continuous metric with the values between 0 and 1—pullback pressure gradient (PPG) index which quantifies hyperemic PPG and discriminates focal from diffuse CAD (21). The PPG index values ≥0.65 are considered hemodynamically focal CAD, values ≤0.47 diffuse CAD, whereas values between 0.47 and 0.65 combined CAD. In the review article, Ilic et al. proposed new algorithm that incorporates iFR-pullback, FFR-pullback and PPG index (<0.40 = focal CAD; >0.70 = diffuse CAD) into serial stenoses assessment integrating all three indices. iFR- and FFR-pullback estimate pressure drop accross each lesion, while PPG index evaluates the distribution of CAD in the entire artery, assuring adequate treatment for focal lesions while avoiding unnecessary treatment for diffuse disease that has no clinical benefit. However, this strategy needs to be evaluated in clinical trials regarding both short- and long-term clinical outcomes.

In the prospective randomized study, Ilic et al. investigated the effect of anti-ischemic drug trimetazidine (TMZ) in patients with chronic coronary syndrome and positive non-invasive stress test, given before elective PCI, on microcirculation using invasively measured index of microvasculatory resistance (IMR). Using contemporary invasive physiological index for the first time, they found that TMZ pretreatment improved coronary microvascular function and prevented PCI-related microvascular impairment by increasing postprocedural-FFR and lowering postprocedural-IMR. It implies that TMZ may be beneficial for patients with ischemia and non-obstructive coronary arteries (INOCA), but further studies are needed.

Finally, Giga et al. have presented noninvasive study with transthoracic Doppler echocardiography (TDE) in which it has been shown that diastolic deceleration time (DDT) measured one months after successfully reperfused first anterior myocardial infarction (MI) is a useful tool for the assessment of microcirculatory function. They have shown that DDT <886 msec in the chronic phase of MI has a good diagnostic accuracy for the identification of large fixed perfusion abnormalities as assessed by SPECT (AUC 84%), with a sensitivity and specificity of 89% and 62%, respectively. The presence of steep decelarition in diastolic coronary flow velocity (CFV) with short DDT reflect the presence of microvascular injury and/or obstruction with the subsequent increase in microvascular resistence. Accordingly, the shorter DDT is related to a larger infact size, adverse left ventricle remodeling, impaired global systolic function and contractility, and poor prognosis. Further clinical trials are required to confirm these findings.
